# RAS modulation prevents progressive cognitive impairment after experimental stroke: a randomized, blinded preclinical trial

**DOI:** 10.1186/s12974-018-1262-x

**Published:** 2018-08-13

**Authors:** Heba A. Ahmed, Tauheed Ishrat, Bindu Pillai, Abdelrahman Y. Fouda, Mohammed A. Sayed, Wael Eldahshan, Jennifer L. Waller, Adviye Ergul, Susan C. Fagan

**Affiliations:** 1 Program in Clinical and Experimental Therapeutics, Charlie Norwood VA Medical Center and University of Georgia College of Pharmacy, HM Bldg., 1120 15th St, Augusta, GA 30912 USA; 2 Department of Anatomy and Neurobiology, College of Medicine, Neuroscience Institute, University of Tennessee Health Science Center, Memphis, TN USA; 3 Department of Physiology, Augusta University, Augusta, GA USA; 4 Department of Neurology, Augusta University, Augusta, GA USA; 5 Department of Biostatistics and Epidemiology, Augusta University, Augusta, GA USA

**Keywords:** Angiotensin modulators, Cognitive-impairment, Hypertension, Stroke

## Abstract

**Background:**

With the aging population, the prevalence and incidence of cerebrovascular disease will continue to rise, as well as the number of individuals with vascular cognitive impairment/dementia (VCID). No specific FDA-approved treatments for VCID exist. Although clinical evidence supports that angiotensin receptor blockers (ARBs) prevent cognitive decline in older adults, whether ARBs have a similar effect on VCID after stroke is unknown. Moreover, these agents reduce BP, which is undesirable in the acute stroke period, so we believe that giving C21 in this acute phase or delaying ARB administration would enable us to achieve the neurovascular benefits without the risk of unintended and potentially dangerous, acute BP lowering.

**Methods:**

The aim of our study was to determine the impact of candesartan (ARB) or compound-21 (an angiotensin type 2 receptor––AT2R––agonist) on long-term cognitive function post-stroke, in spontaneously hypertensive rats (SHRs). We hypothesized that AT2R stimulation, either directly with C21, or indirectly by blocking the angiotensin type 1 receptor (AT1R) with candesartan, initiated after stroke, would reduce cognitive impairment. Animals were subjected to a 60-min transient middle cerebral artery occlusion and randomly assigned to either saline/C21 monotherapy, for the full study duration (30 days), or given sequential therapy starting with saline/C21 (7 days) followed by candesartan for the remainder of the study (21 days). Outcome measures included sensorimotor/cognitive-function, amyloid-β determination, and histopathologic analyses.

**Results:**

Treatment with RAS modulators effectively preserved cognitive function, reduced cytotoxicity, and prevented chronic-reactive microgliosis in SHRs, post-stroke. These protective effects were apparent even when treatment was delayed up to 7 days post-stroke and were independent of blood pressure and β-amyloid accumulation.

**Conclusion:**

Collectively, our findings demonstrate that RAS modulators effectively prevent cognitive impairment after stroke, even when treatment is delayed.

**Electronic supplementary material:**

The online version of this article (10.1186/s12974-018-1262-x) contains supplementary material, which is available to authorized users.

## Background

Vascular cognitive impairment/dementia (VCID), which defines alterations in cognition attributable to cerebrovascular causes regardless of pathogenesis or severity [1, 2], is an extremely common and debilitating, albeit understudied, long-term complication of stroke, impacting nearly 30–40% of all stroke survivors [3]. The severity of VCI can range from subtle cognitive deficits to overt dementia [4]. At a time when stroke mortality is going down, the rate of stroke-related dementia almost doubled between 1990 and 2000 [5]. Unlike sensorimotor deficits, which are often maximal in the hours following stroke and tend to gradually improve, cognitive deficits increase over time, in both patients [6] and experimental animal models [7]. Until recently, it was thought that vascular dementia develops in a step-wise fashion, due to repeated ischemic events, but recent data from a large, NIH-funded, epidemiologic trial proved that patients often experience a slowly progressive cognitive decline after a single-stroke lesion, in addition to an acute change [3, 5]. This continuous deterioration occurs even in the absence of any new stroke lesions [8].

Unfortunately, no FDA-approved treatments exist for VCID, increasing the urgency for treatment development in this area, as identifying drug therapies that target the pathological processes associated with progressive VCID continues to be a vital unmet need [9].

One of the most important risk factors for development and progression of cognitive impairment and dementia is hypertension [5, 10]. Hypertension, which is associated with vascular dysfunction, has been established to increase the risk of dementia both independently and by increasing the risk of stroke. In fact, hypertension alone can predict the development of dementia in nearly 60% of subjects with executive dysfunction [11].

Even with Alzheimer’s disease (AD), vascular dysfunction may be of greater importance than amyloid accumulation in terms of influencing the disease course, especially in older individuals [5, 7]. Likewise, vascular brain injury is now considered a very early, and perhaps initial, pathogenic mechanism not only in VCID but also in AD [8].

Angiotensin II (Ang II), the major effector/ligand of the intrinsic brain renin angiotensin system (RAS), is a neuropeptide produced mostly by glial cells in the CNS [12] and targets the brain’s two major receptor subtypes AT1R and AT2R, both primarily expressed in the vasculature [13]. Hyperactivation of brain AT1R is responsible for the damaging effects associated with RAS as it promotes vasoconstriction, reduces CBF, and increases oxidative stress and vulnerability to ischemia, in addition to promoting vascular and tissue inflammation and exacerbating neurodegeneration [12].

Our group has shown that ARBs, by indirectly stimulating brain AT2Rs, are both vascular and neuroprotective after ischemic stroke [14]. These agents also effectively preserved cognitive function and prevented the development/progression of VCID in aged animals with chronic cerebral hypoperfusion [15]. Stimulation of the AT2R has emerged as a novel therapeutic strategy in vascular and CNS diseases by virtue of its anti-inflammatory, neuroprotective, and tissue-regenerating properties [6, 8]. In our recent large, randomized preclinical trial of the AT2R agonist, compound 21, although neuroprotection was modest, we found a consistent benefit of C21 on cognition after stroke [16]. The purpose of this investigation was to determine the impact of RAS modulation on the development of cognitive impairment after stroke in hypertensive animals, as a proposed model of VCID.

Clinical trial evidence supports that ARBs, when used for the treatment of hypertension, prevent cognitive and functional decline in older adults and lower the risk of dementia and AD [2, 11, 17]. Unfortunately, these agents also reduce blood pressure (BP), an AT1R effect which is undesirable in the acute stroke period (first 7 days) [18]. This is because during the acute phase post-stroke, a fall in BP may lead to an excessive reduction in cerebral perfusion, and is especially true with long-standing hypertension, due to associated impairments in cerebrovascular autoregulation. Moreover, based on the findings of the large clinical (SCAST) study, significant acute reductions in BP with candesartan should be avoided, to allow perfusion through collateral vessels and optimize salvage of penumbral tissue post-stroke [18]. We hence decided to initiate treatment with candesartan, only after the first 7 days (replacing C21 or saline). This approach was taken in order to harness the neurovascular benefits of AT2 receptor stimulation with C21, in the acute phase, while avoiding the early antihypertensive effects of candesartan, and to model what could be an effective strategy in clinical practice.

## Methods

### Experimental design, animals, and treatment

**Fig. 1:**
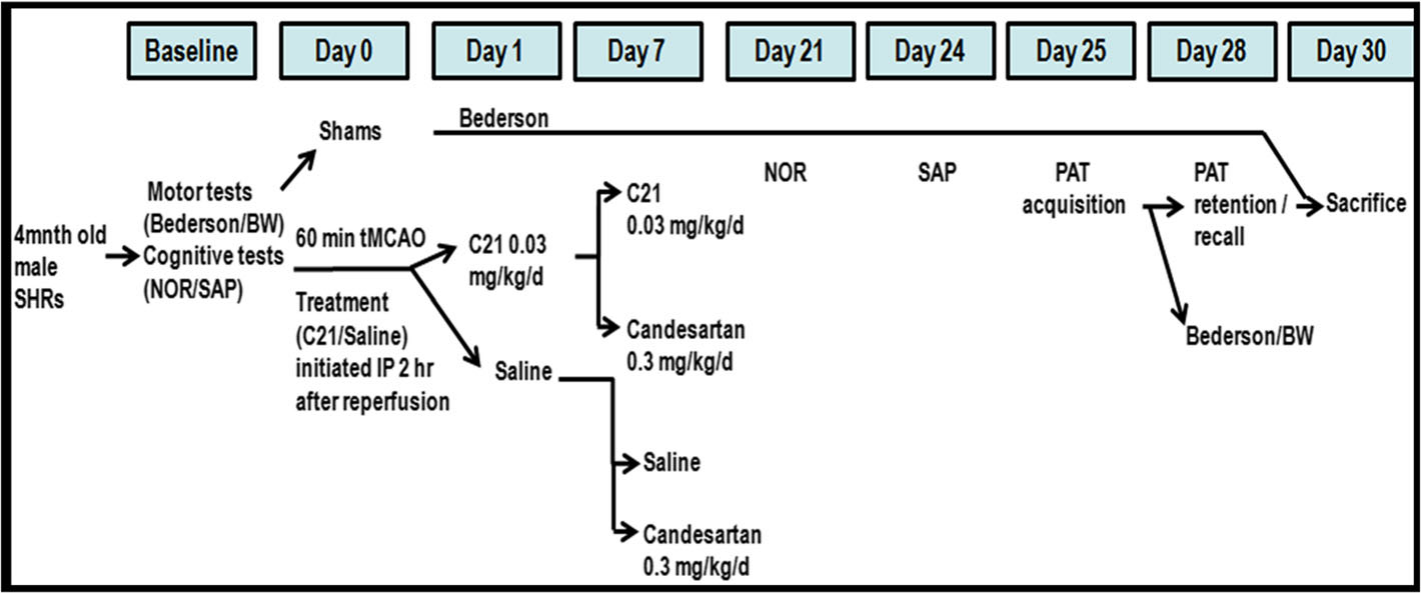
Schematic time-line and description of the experimental design. This was a long-term 2 × 2, randomized, double-blind preclinical trial aimed to study the impact of RAS modulation, after stroke, on long-term cognitive function in spontaneously hypertensive rats (SHRs)

As summarized in Fig. 1, this was a 2 × 2, randomized, double-blind (investigators and statistician), preclinical trial. In total, we used 41 young adult (4 months old), male SHRs, weighing 290–300 g, obtained from Charles River Laboratories. Of these animals, 33 were subjected to a 60-min temporary middle cerebral artery occlusion (tMCAO) and 8 animals were exposed to sham surgery. Animals were housed in a pathogen-free environment with free access to food and water on a 12-h light/dark cycle before and after surgery. All animals exposed to 60 min of temporary middle cerebral artery occlusion (tMCAO) were randomized, after reperfusion, using a random number generator, to one of 4 groups. Animals received either C21 (0.03 mg/kg/day) (VicorePharma, GӦteborg, Sweden) or saline, initiated IP 2 h after the onset of reperfusion, and continued for a total of 7 days, after which the animals were either switched to a low dose of candesartan (0.3 mg/kg) IP or continued with their initial treatments (C21 or saline), daily for the remainder of the study. Treatments were prepared in identical syringes by an investigator not involved in the administration or assessment of the animals and unblinding only occurred after all data was collected and analyzed. The entire set of data was reported, and included in the final data analyses, for each and every animal that survived and completed all the necessary behavioral tests in the study, without exclusion of any animals/data points. Therefore, all the animals that started the study, with the exception of the 3 animals that did not complete any of the behavioral tests (one animal died at 24 h, the other 2 did not survive past 9 days post-stroke due to excessive weight loss) successfully completed the study and were included in the final analyses. Animal experiments were conducted and  reported in accordance with the *Animal Research: Reporting of In Vivo Experiments (ARRIVE)*, *Stroke Therapy Academic Industry Roundtable* (*STAIR*), and Reporting Guidelines for Risk Models (RiGoR) guidelines [13–15].

### Focal cerebral ischemia

The skin on the cervical region was incised to access the common carotid artery, under anesthesia maintained at 3% isoflurane mixed with 30% oxygen and 70% nitrogen. Ischemia/reperfusion injury was achieved by occluding the middle cerebral artery (MCA) using 4–0 silicon-coated nylon suture with rounded tip, which was inserted into external carotid artery and advanced through internal carotid artery until it blocked the origin of the MCA. Successful MCAO was confirmed by the presence of hemiparesis prior to reperfusion. After the 60-min occlusion, animals were re-anesthetized and sutures removed to allow reperfusion of ischemic brain areas. Animals were returned to their home cages after MCAO with easy access to food and water [14].

### Assessment of functional outcome

#### Body weight

Weight monitoring gives an independent and unambiguous measure of an animal’s overall health and welfare [19]. For our studies, animals were weighed before surgery and then daily after stroke until the day of sacrifice.

### Blood pressure monitoring

To study the effect of different doses of C21 (0.1, 0.03, 0.06 mg/kg/day) and candesartan (0.3, 1 mg/kg/day) on BP, a separate set of animals (6 adult male SHRs) was monitored using continuous BP telemetry. Blood pressure was monitored before tMCAO, after occlusion and following reperfusion, using continuous BP telemetry with transmitters (Data Sciences International, St Paul, MN, USA). These were implanted in rats, under 3–5% isoflurane inhalation anesthesia, as reported previously according to the manufacturer’s specifications [20]. In brief, a midline incision was performed to expose the abdominal aorta, which was shortly occluded to allow insertion of the transmitter catheter, which was secured in place using tissue glue. The incision was closed using non-absorbable suture (3–0). Rats were returned to their individual cages and allowed to recover from surgery for 10 days. By placing cages on top of the telemetry receivers, arterial pressure waveforms were continuously recorded throughout the study. These animals were later treated with increasing doses of C21 (0.01, 0.03, 0.06 mg/kg/day). Each dose was given for three consecutive days and followed up by a 24-h washout period before initiation of the next dosage increment. After the final washout, the animals were treated with two different doses of candesartan 0.3 and 1 mg/kg/day (separated by a 48-h washout period). The following week animals underwent a 60-min tMCAO and were given saline/C21 (0.03 mg/kg/d) for 7 days followed by candesartan (0.3 mg/kg/d) for the remainder of the monitoring period [20].

### Neurobehavioral testing

All neurobehavioral tests were conducted, recorded, and analyzed in a blinded manner.

#### Sensorimotor testing

To assess sensorimotor function, animals underwent Bederson and beam walk tests at baseline and at day 1 and day 28 post-stroke.

##### Bederson score

Animals were assigned a score from 0 to 3, given one point for each of the following: forelimb flexion, diminished resistance to lateral push, and contralateral circling with lower scores indicating better performance [21].

##### Beam walk

Animals were  placed on a horizontal beam (60 cm long, 4.5 cm wide) for 60 s and assigned a score from 0 to 6 as follows: balances on the beam with a steady posture = 0, grasps side of the beam = 1, hugs the beam with 1 limb falling = 2, hugs the beam with two limbs falling = 3, falls off the beam within 40–60 s = 4, falls off the beam within 20–40 s = 5, and falls off the beam in less than 20 s = 6 [14].

#### Memory, cognitive, and neurobehavioral testing

Cognitive tests were performed at baseline and prior to sacrifice at 30 days. In a subset of animals treated with either saline or C21, cognition (novel object recognition––NOR) was tested at 14 and 21 days. The NOR and spontaneous alternation performance (SAP) tests were performed to evaluate non-spatial and spatial working memories [22–25], respectively, while the passive avoidance test (PAT) assessed associative learning and reference memory [26, 27].

#### The novel object recognition (NOR) test

The NOR test was performed to evaluate non-spatial working memory related to frontal-subcortical circuits [22]. This test was based on the spontaneous tendency of animals to interact with a novel object more than a familiar one, and  consisted of 2 trials separated by a retention period and preceded by a habituation phase. The habituation phase was conducted on 2 separate days, before the start of the test, to allow animals to acclimate to their arena, which consisted of placing them in  an empty standard size box for 15 min. On the designated test day, animals were first subjected to an acquisition/sample trial, where the animal is presented with 2 identical (sample) objects and allowed to explore for 10 min [28, 29]. Following sample object exposure, the animal was returned to its home cage for a 1-h retention period. The 2nd preference trial/test session (5 min), which follows the retention period, was conducted in the same manner as the 1st trial, except that a new/novel object replaces one of the familiar/sample objects. The arena and objects were cleaned after each session with 70% ethanol. The time spent in exploring each object during the preference trial/test session was recorded and the discrimination index, which is the difference in exploration time for the objects divided by total time of exploration, was calculated. The discrimination index (DI) and the recognition index (RI), which is the time spent exploring the novel object relative to the total time of exploration, were taken as indicators of working memory [28, 30].

$$ \mathrm{Discrimination}\ \mathrm{index}\ \left(\mathrm{DI}\right)=\left({T}_N-{T}_F\right)/\left({T}_N+{T}_F\right) $$

$$ \mathrm{Recognition}\ \mathrm{index}\ \left(\mathrm{RI}\right)={T}_N/\left({T}_N+{T}_F\right) $$

- ▪ Time spent interacting with the familiar object (**T**_**F**_)
- ▪ Time spent interacting with the novel object (**T**_**N**_)

All animals passed the required exploratory criteria, exploring  the objects for > 4 out of the 5 min. All the objects to be discriminated were unified between animals and chosen according to recommendations of Heyser and Chemero; they were symmetrical and transparent, and  made of odorless, durable, and easy to clean plastic and glass [24].

#### The spontaneous alternation performance (SAP) test

The SAP test evaluates spatial working memory using a spontaneous alternation paradigm, which also takes advantage of rodents’ natural preference for environmental novelty. Assessment was performed using a typical Y-maze apparatus, with 3 equidistant arms of similar size and dimensions (50 × 10 × 19 cm) each having acrylic walls adorned with specific motifs, serving as visual cues to aid in spatial navigation. After introduction to the center of the maze, the animal was allowed to freely explore all 3 arms. Over the course of multiple arm entries, the subject should show a tendency to enter the least recently visited arm and thus tend to alternate visits between the 3 arms. The number of arm entries and the number of triads are recorded in order to calculate the percentage of alternation. An entry occurs when all four limbs are within the arm. The SAP was assessed by scoring the pattern of entries into each arm during the 5-min test. Alternations were defined as successive entries into each of the 3 arms as on overlapping triplet sets (i.e., ABC, BCA). %SAP is defined as the ratio of actual (= total alternations) to possible (= total arm entries-2) number of alternations × 100 [23].

### The passive avoidance test (PAT)

The inhibitory avoidance (IA) test (commonly referred to as passive avoidance test) was used to assess aversive associative learning and related reference memory. This test is designed to condition against an animal’s urge to enter a dark chamber or explore a novel compartment, when exposed to an aversive stimulus [25]. The animal must therefore “actively inhibit” its desire and withhold the response, to re-enter a dark or novel compartment, after it has experienced an aversive stimulus in that location. For this test, one of the compartments of the Y-maze was equipped with a metal floor connected to an electric circuit box, adjusted to deliver brief, moderate intensity electric shocks (3 s duration, 0.6–1 mA). For the acquisition trial, the shock compartment/arm was blocked and the animal placed in one of the “safe” arms and allowed 10 min to explore the 2 open arms. Upon completion of 10 min, the door blocking the shock arm was opened allowing the animal to enter. Once the animal had fully entered the shock arm, its initial latency was recorded and it received a brief electric shock before being returned to its cage. After a 72-h retention period, the test trial was conducted. This was performed in a manner similar to that of the acquisition trial except that the foot shock was omitted and all 3 arms were accessible to the animal from the start. The difference, between training and test sessions, in latency for entering into the desired compartment (shock arm) was used as a measure of retention. This latency was recorded for up to 300 s, as the index of long-term aversive associative memory consolidation [20–22].

### Animal sacrifice and tissue collection

At day 30, animals were anesthetized with IP ketamine/xylazine and transcardially perfused with 300 ml of ice cold PBS. Animals were decapitated, and their brains collected. Sections A and B, from brain matrix, were snap frozen and kept for ELISA. The remaining brain tissue was immersed in 10% formalin (Fischer Scientific, Waltham, MA, USA) for 48 h and then transferred to a 30% sucrose solution until taken for sectioning [14].

### Immunofluorescent staining and confocal microscopy

Frozen brain sections (5 μm thick) were processed and stained following a standard technique as previously described [14]. Briefly, sections were heated in 10 mM sodium citrate buffer (pH 6) and then washed in TBS plus 0.025%/Triton X-100 with gentle agitation, blocked for 2 h in 10% normal goat serum/1% BSA, and incubated overnight at 4 °C with Alexa fluor 488 anti-amyloid-β1–16 (6E10; 1:1000; Biolegend) or anti-ionized calcium-binding adapter molecule-1 (Iba-1; 1:1000; Wako). After washing, slides were incubated with their appropriate fluorophore-conjugated goat secondary antibodies (1:1000; Abcam) for 1 h at room temperature and washed and cover-slipped with Vectashield mounting medium (Vector Laboratories, Burlingame, California, USA). Images were taken using Zeiss LSM 510 confocal microscope (Carl Zeiss).

### Histopathologic analyses and quantification

The number of activated microglia, determined by Iba-1 staining, and degree of apoptotic cell death, determined by the ApopTag fluorescein in situ apoptosis detection kit [S7110, Chemicon (Millipore), Temecula, CA], in the ischemic borderzone region of the ipsilateral hemisphere for each brain section, was quantified using ImageJ software (NIH), in 3 different fields per section, digitized with a × 20 objective lens [14]. To quantitatively characterize microglia morphology, we used the particle measurement feature in ImageJ to automatically measure the 2D area, perimeter and Feret’s diameter which is the greatest distance between any two points along the cell perimeter. This is a measure of cell length and serves partly as an indicator of microglial phenotype. We also evaluated other parameters including circularity and transformation index, [perimeter of cell (μm)]^2^/4π [cell area(μm^2^)], which reflect the degree of process extension and serve as established indicators categorizing microglial ramification status [23–25].

### Protein expression

Quantitative determination of Aβ_1–42_ concentrations in cortical and hippocampal lysates were carried out, using a rat specific sandwich amyloid-β ELISA kit (Wako, USA), according to manufacturer’s protocol.

### Endothelial cell culture and treatments

#### Amyloid-β (Aβ_1–42_) preparation

Synthetic Aβ_1–42_ was prepared to form soluble oligomeric peptides for endothelial cell culture studies, according to manufacturer’s recommendations (AnaSpec, San Jose, CA, USA). The end product was a clear homogenous preparation, free from insoluble aggregates. Absence of aggregates or fibrils was confirmed under microscope.

### Oxygen and glucose deprivation (OGD)

Human cerebral microvascular endothelial cells (hCMEC/D3), generously donated by Dr. J Zastre (University of Georgia), were cultured to confluence in normal oxygen/glucose, MCDB-131 complete medium (VEC Technologies, Rensselaer, NY). To mimic in vivo ischemia-reperfusion, these cells were subjected to 8 h OGD by incubation in glucose-free DMEM (GIBCO), at 37 °C in a hypoxia chamber (ProOx model C21; BioSpherix, Lacona, NY) with an O_2_ concentration of 1% and CO_2_ of 5%, followed by 16 h of standard normoxic conditions (95% air, 5% CO_2_) and replacing the glucose-free DMEM with serum-free Eagle’s minimum essential medium (EMEM; American Type Culture Collection, Manassas, VA, USA), in the presence or absence of Aβ_1–42_ (100 nM) with or without treatment, using angiogenic concentrations of C21 (100 nM, 1000 nM) [14] or candesartan (1 μg/ml, 10 μg/ml), before being subjected to the MTT cell viability assay [31].

### Mitochondrial tetrazole test (MTT)

The extent of cell death was determined indirectly by the MTT cell viability assay. This test is based on the ability of living, metabolically active, cells to reduce a yellow tetrazole compound, 3-(4,5-dimethylthiazol-2-yl)-2,5-diphenyltetrazolium bromide, into an insoluble purple formazan product, with an absorbance maximum at 570 nm, measured using a microplate reader (Synergy HT, BioTek, VT, USA). Intensity of color formation/absorbance serves as a marker of cell viability [32].

### Statistical analysis

All behavioral analysis was performed using SAS 9.4 by a blinded statistician, and statistical significance was assessed using an alpha level of 0.05, unless otherwise noted. Repeated measures mixed models were used to examine differences in outcomes between the five groups (SHAM, Stroke Saline, Stroke C21, Stroke Candesartan, and Stroke C21 + Candesartan) over days (0 and 21 days for NOR measures; 25 and 28 days for PAT). For each outcome, the mixed model contained fixed effects of group, day, and the two-factor interaction between group and day. Animal nested within group was considered a random effect. As there were only two measurement times, an unstructured correlation structure was used to model the correlation between days. The F-test for the two-factor interaction between group and day was the statistical test of interest and, if statistically significant, indicates that the changes in the outcomes over time are different in the five groups. Post hoc, pair-wise comparisons between groups over time were performed using a Bonferroni adjustment to the overall alpha level. To examine differences in weight over a 31-day period (from pre-surgery, 0 to 30 day following surgery) between Candesartan and C21 groups (Candesartan/C21, Candesartan/Saline, C21/Saline, Saline/Saline), a quadratic growth curve model was utilized. Fixed effects in the model included Candesartan and C21. Day was considered a continuous variable. Random effects included the intercept, linear day term, and the quadratic day^2^ term. An unstructured variance co-variance structure provided the best model fit. The interaction between Candesartan, C21 and the quadratic day term and the interaction between Candesartan and the quadratic day term which were not statistically significant were removed from the model. Statistical significance for differences between groups for specific days (0, 1, 7, 10, 30) were examined using the estimated least squares means from a repeated measures mixed model and denoted by *P < 0.0001, using a Bonferroni adjustment to the overall *α*-level for the number of post hoc pair-wise comparisons performed within group between measurement days and within days between groups was used to control for the number of tests performed. Differences between groups in immunostaining experiments were examined using ANOVA followed by Tukey test for post hoc analysis using GraphPad prism software (5.1). Exact power calculations were not performed in this study as the effect size could not be accurately estimated due to lack of previous studies utilizing these specific groups.

## Results

### Compound 21 had no effect on BP in SHRs before or after stroke

**Fig. 2:**
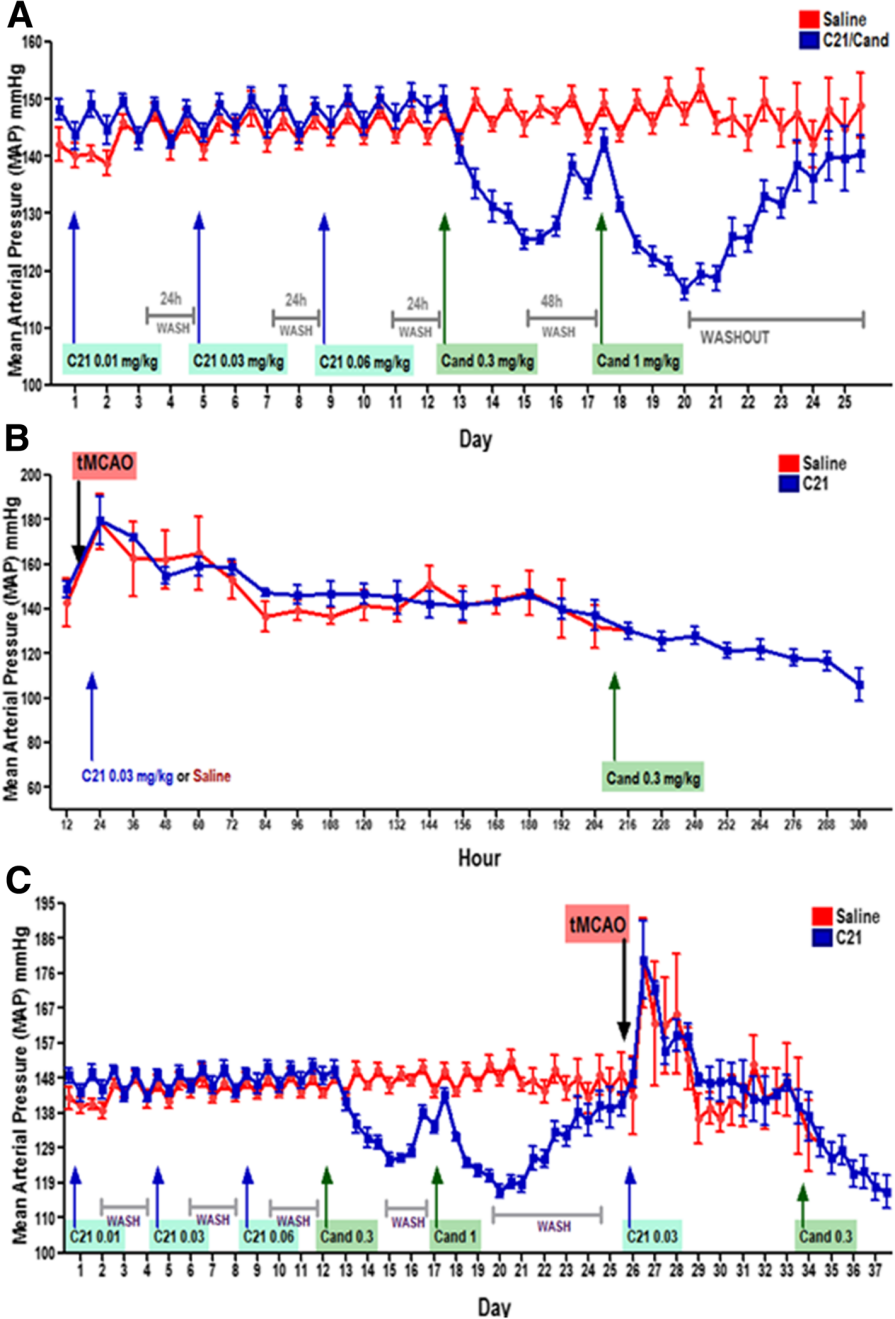
Compound 21 had no effect on blood pressure in SHRs after a 60-min tMCAO. **a** Mean arterial pressure (MAP), as measured by telemetry, showed no effect of C21 on BP, in unstroked animals. **b** There was an increase in MAP from baseline after transient middle cerebral artery occlusion (tMCAO) and this was not affected by C21 administration 2 h after reperfusion, and the average BP trend was similar for both C21 and saline-treated animals. **c** Figure depicting complete MAP trend, before and after tMCAO, with the effect of treatments administered at different points in time

Stroke increased BP from baseline by over 30 mmHg in SHRs after a 60-min tMCAO. This was reduced slightly after reperfusion, but remained elevated for at least 24 h. This is similar to the trend we had previously observed after a 3-h tMCAO in both SHRs [20] and Wistar rats [14]. In SHRs, C21 treatment had no effect on BP compared to saline either before or after stroke (Fig. 2). Doses of 0.03 mg/kg/day C21 and 0.3 mg/kg/d candesartan were chosen based on our preliminary findings and previous publications [8, 33]. These doses were shown to have superior cognitive benefits than other doses when administered intraperitoneally, with little or no effect on BP. Moreover, C21 when administered daily, starting at 2 h after tMCAO and reperfusion, significantly ameliorated weight loss at day 7, compared to saline-treated controls (Additional file 1), with animals showing most rapid weight recovery, between days 7 and 30, when treatment was followed with candesartan.

### All animals experienced complete motor recovery after transient ischemic stroke

**Fig. 3:**
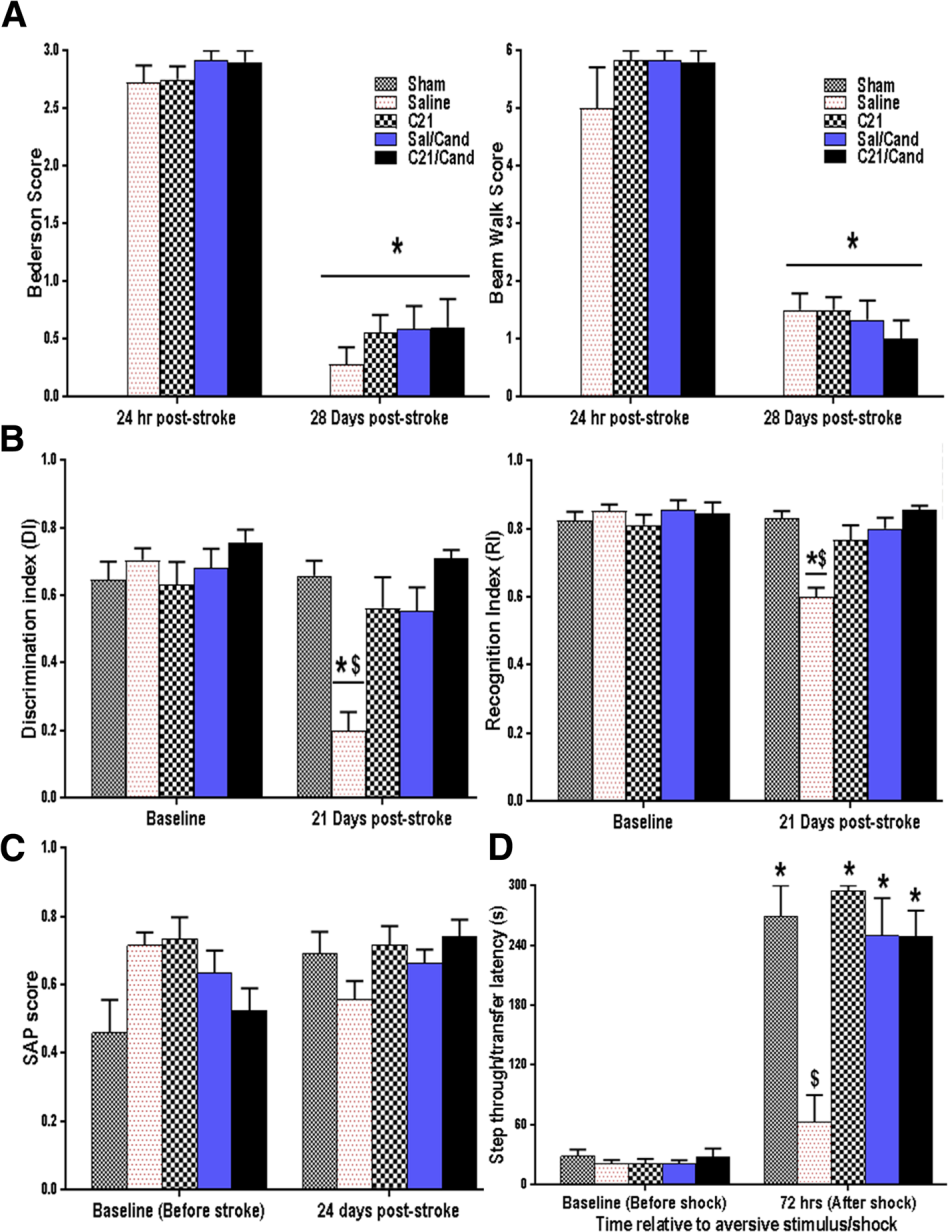
RAS modulation improved learning and preserved spatial, non-spatial working, and long-term reference memories despite lack of effect on long-term motor recovery post-stroke. **a** All animals showed a significant recovery of sensorimotor function, as measured by Bederson (C21 × candesartan × time interaction *F*_(1,48)_ = 0.22, *P* = 0.6384) and Beam walk (C21 × candesartan × time interaction *F*_(1,31)_ = 0.41, *P* = 0.5278), at 28 days post-stroke, irrespective of their assigned treatment group. On the other hand, **b** for the novel object recognition (NOR) test, saline-treated animals showed a significant reduction in discrimination index (DI) and recognition index (RI), compared to their baseline values as well as to all other groups post-stroke, while sham and C21/candesartan-treated animals retained their ability to recognize the novel object during the preference trial indicating preserved non-spatial working memory (group × time interaction *F*_(1,30)_ = 14.58, *P* < 0.0001). **c** Spontaneous alternation performance (SAP) was also noticeably better for these animals with plots of the means from baseline to 24 days showing distinctive changes within each group, shams, and candesartan/C21-treated groups having either no change from baseline or an increased SAP, while the saline group showed a clear decrease in SAP from 0 to 24 days indicating worsened spatial working memory (C21 × candesartan × time interaction *F*_(1,17)_ = 0.12, *P* = 0.7314). **d** On the passive avoidance test (PAT), sham animals and those treated with C21/candesartan showed significantly enhanced step-through latency, indicating better memory retention and recall of the association between properties of the chamber and the foot shock, compared to their saline-treated counterparts. (group × time interaction *F*_(1,39)_ = 19.00, *P* < 0.0001). For all tests *n* = 5–10 animals/group, error bars indicate SEM. Statistical significance is denoted by *P < 0.0001 for post hoc pair-wise comparisons from baseline and **#***P* < 0.0001 for post hoc pair-wise comparisons “between groups” post-stoke, using the Bonferroni-adjusted alpha

Hypertensive animals subjected to a 60-min tMCAO showed visible sensorimotor deficits within the first few hours of stroke. These deficits, marked with high Bederson and beam walk scores (Fig. 3a), were pronounced at 24 h post-stroke, and all treatment groups showed similar recovery at 28 days post-stroke.

### RAS modulation effectively prevented cognitive decline post-stroke

Sham animals did not demonstrate cognitive decline at 28 days. Despite complete recovery of sensorimotor function, the saline-treated animals demonstrated a continuous decline in cognition from days 14 to 28 (Additional file 2). Chronic administration of C21, or candesartan, prevented this decline, even when treatment was initiated at 7 days after the ischemic insult. The groups treated with C21 and candesartan demonstrated superior performance on the NOR test, compared to saline-treated animals (Fig. 3b). In fact, most of the C21 and candesartan-treated animals showed discrimination and recognition indices that were not much different from their original baseline pre-stroke values. These animals also tended to display more efficient SAP, indicating better spatial working memory (Fig. 3c), and exhibited significantly higher transfer/step-through latencies on the PAT retention as compared to the acquisition trials (Fig. 3d). This not only indicates an intact reference memory, with retention of the aversive event, it also signifies effectual associative learning and recall of the connection between properties of the chamber and the foot shock. The saline-treated animals, however, exhibited compromised learning and memory as they failed to show any meaningful increase in transfer/step-through latency on the retention trial.

### C21 prevented accumulation of Aβ_1–42_ in the hippocampus of SHRs post-stroke

**Fig. 4:**
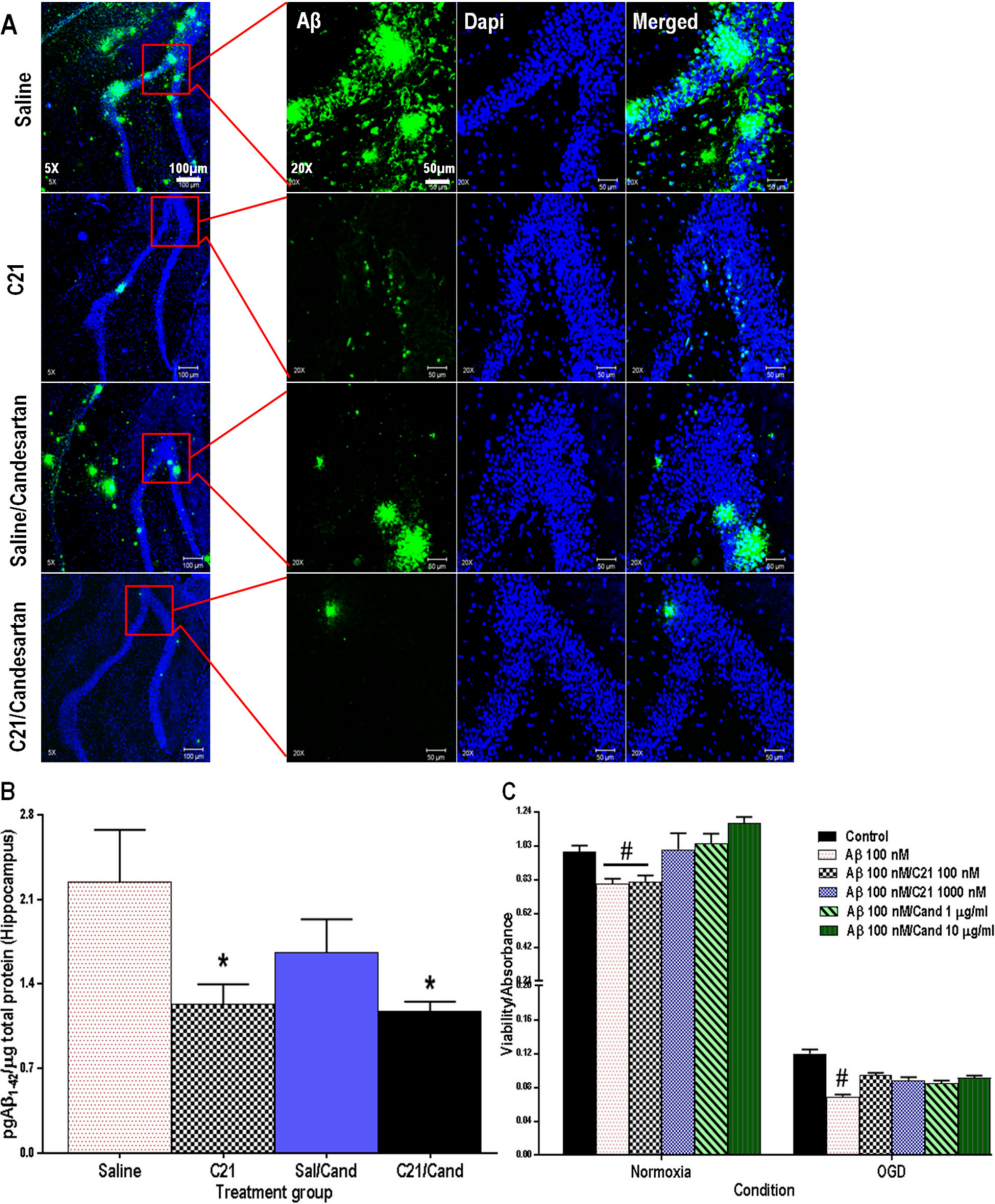
RAS modulation prevented Aβ_42_-mediated cytotoxicity in HBECs and C21 prevented hippocampal Aβ_42_ accumulation in SHRs post-stroke. **a** Representative 5× and 20× immunofluorescent Aβ/green-stained images of SHR hippocampus with blue/dapi nuclear staining, scale bar represents 100 and 50 μm for 5× and 20× images, respectively. **b** An Aβ_1–42_ ELISA analysis showed that animals treated with C21, for the first 7 days, had markedly lower hippocampal concentrations of Aβ_1–42_ at 30 days post-stroke than those treated with saline. (C21 × candesartan interaction *F*_(1,18)_ = 1.30, *P* = 0.2719, *n* = 5–10 animals/group. **c** Cell viability (MTT conversion) was significantly reduced in cultured HBECs incubated with Aβ_1–42_ compared with untreated controls, under similar conditions. This cytotoxicity was prevented when cells were co-treated with candesartan and higher dose C21 (condition by Aβ/C21/candesartan group interaction *F*_(5,89)_ = 5.68, *P* = 0.0002, *n* = 6–12 wells/group, error bars indicate SEM). Statistical significance for post hoc comparisons between groups using Tukey’s multiple comparison procedure are denoted by *P < 0.05 to indicate a difference from saline and **#***P* < 0.05 to indicate a difference from all other treatment groups

Aβ accumulation was assessed using an Aβ_1–42_ specific ELISA kit and confirmed visually by immunofluorescent staining, illustrating dense core plaques in the hippocampus (Fig. 4a). Animals treated with C21 for the first 7 days after ischemic stroke had markedly lower hippocampal concentrations of Aβ_1–42_ at 30 days post-stroke than those treated with saline (Fig. 4b). Similar results were found in the cortical ischemic borderzone (Additional file 3). This finding, although novel, may not be responsible for the cognitive benefits associated with RAS modulators since animals that were switched to candesartan after having been treated with saline for 7 days also displayed preserved cognitive function, despite sustained elevations in hippocampal Aβ_1–42_.

### RAS modulation prevented Aβ_42_-mediated cytotoxicity in HBECs subjected to OGD

Cell viability was significantly reduced in both neurons (Additional file 4) and HBECs incubated with Aβ_1–42_ compared with untreated controls, under similar conditions. This Aβ_1–42_-mediated cytotoxicity was prevented when cells were co-treated with candesartan. This was true with both the 1 and 10 μg/ml doses of candesartan and was apparent under both normoxic and hypoxic conditions. Although both doses of Candesartan (1 μg/ml, 10 μg/ml) were equally effective at preventing Aβ_1–42_-associated endothelial cell death under hypoxic conditions, only the higher dose of C21 (1000 nM) was effective under normoxic conditions (Fig. 4c).

### RAS modulation prevented chronic-reactive microgliosis in SHRs post-stroke

**Fig. 5:**
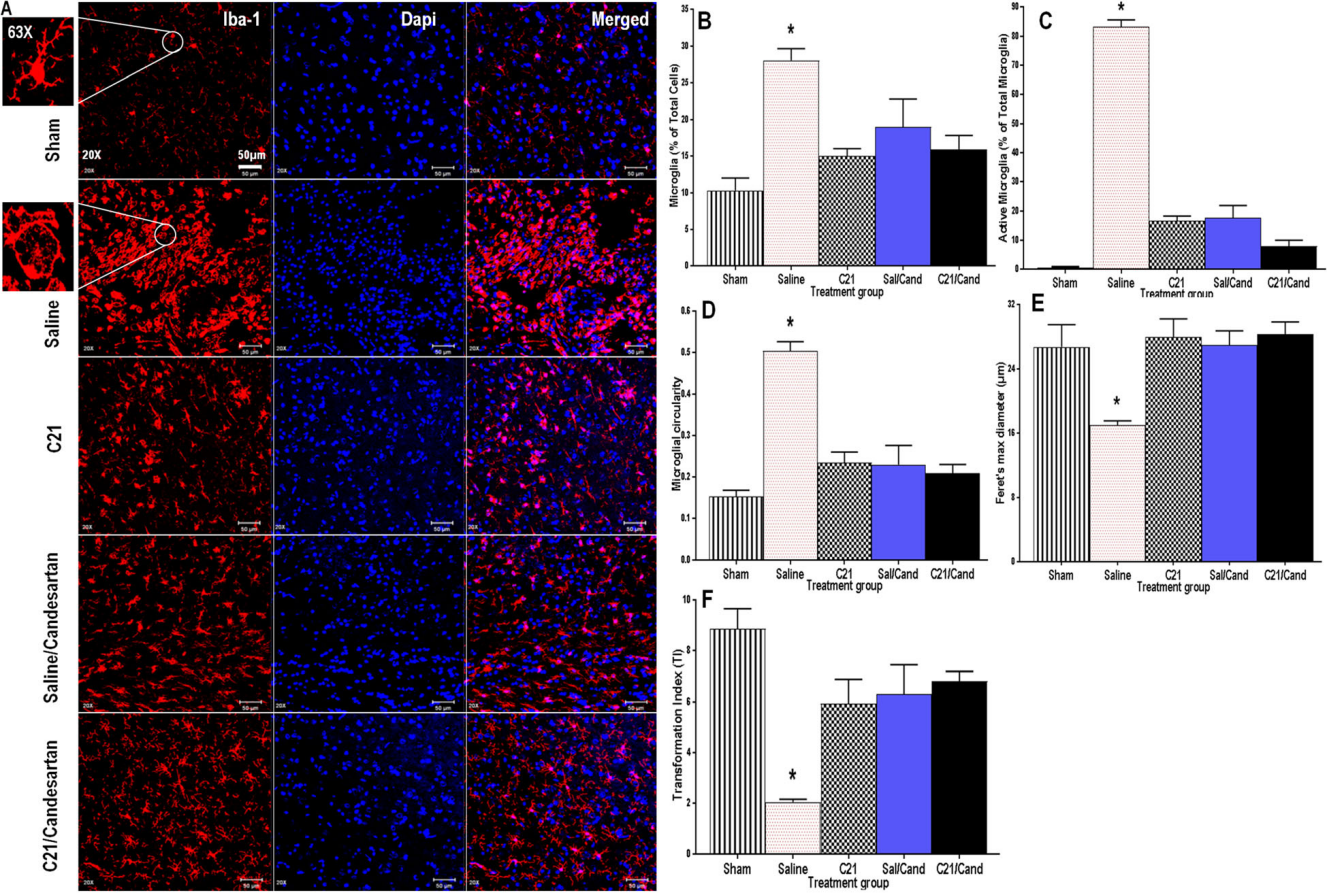
RAS modulation reduced microglial accumulation, offset sustained activation and prevented chronic reactive microgliosis, in SHRs post-stroke. **a** Representative immunofluorescent images depicting microglial morphology/ramification status changes as illustrated Iba-1 positive (red stained) microglia with blue dapi nuclear staining, scale bar represents 50 and 20 μm for 20× and 63× images, respectively. Hypertensive controls (saline) subjected to tMCAO had, in the ischemic hemisphere, a significantly higher proportion of **b** total microglia (*F*_(4,14)_ = 13.18, *P* < 0.05) and **c** active, inflammatory microglia (*F*_(4,14)_ = 249.1, *P* < 0.0001) at 30 days post-stroke relative to shams and C21/candesartan-treated animals. These saline-treated controls also showed **d** increased circularity (*F*_(4,14)_ = 33.52, *P* < 0.0001), **e** reduced Feret’s maximum diameter (*F*_(4,14)_ = 7.314, *P* < 0.05), and **f** reduced transformation index (TI) (*F*_(4,14)_ = 14.02, *P* < 0.0001), all consistent with a transformation of microglia from ramified to reactive amoeboid forms, at 30 days post-stroke relative to both sham and C21/candesartan-treated animals. However, C21 and candesartan-treated animals showed indices not much different from those of shams (*n* = 3–4 animals/group, error bars indicate SEM). Statistical significance for post hoc comparisons between groups using Tukey’s multiple comparison procedure are denoted by *P < 0.05 for differences in proportion of total microglia and Feret’s maximum diameter and *P < 0.0001 for differences in all other parameters. Feret’s (maximum) diameter, a measure of cell length, is the greatest distance between any two points along the cell perimeter [51]. Circularity = 4π × [cell area (μm^2^)]/[cell perimeter (μm)]^2^ is unitless value that ranges from 0 (infinitely elongated)-1 (perfect circle) [51]. Transformation index (TI) = [perimeter of cell (μm)]^2^/4π [cell area(μm^2^)] [51]

Representative immunofluorescent images of microglial morphology/ramification status changes are depicted in Fig. 5a. Hypertensive animals subjected to a 60-min tMCAO showed a significantly higher proportion of total (Fig. 5b) and reactive (Fig. 5c) microglia in the ischemic borderzone at 30 days post-stroke, relative to shams. These microglia displayed classic phenotypic features of an activated, amoeboid morphology, with relatively high circularity (Fig. 5d) and stubby or absent processes, in addition to a low Feret’s maximum diameter (Fig. 5e) and transformation index (TI < 3) (Fig. 5f). This sustained activation (inflammatory), which was prevented in the C21 and candesartan-treated animals, was associated with delayed, albeit significant, cognitive decline. These agents, already established to possess potent anti-inflammatory and antioxidant properties [12], completely abolished chronic reactive microgliosis, known to be the chief source of cytokines and reactive oxygen species that drive progressive neuronal damage. Consistent with a transformation of amoeboid to healthy ramified microglia in these RAS-treated animals, the TI and Feret’s maximum diameter was significantly higher and cell circularity was significantly lower than those of saline-treated animals, values that were not much different from sham animals.

### RAS modulation reduced apoptotic cell death post-stroke

**Fig. 6:**
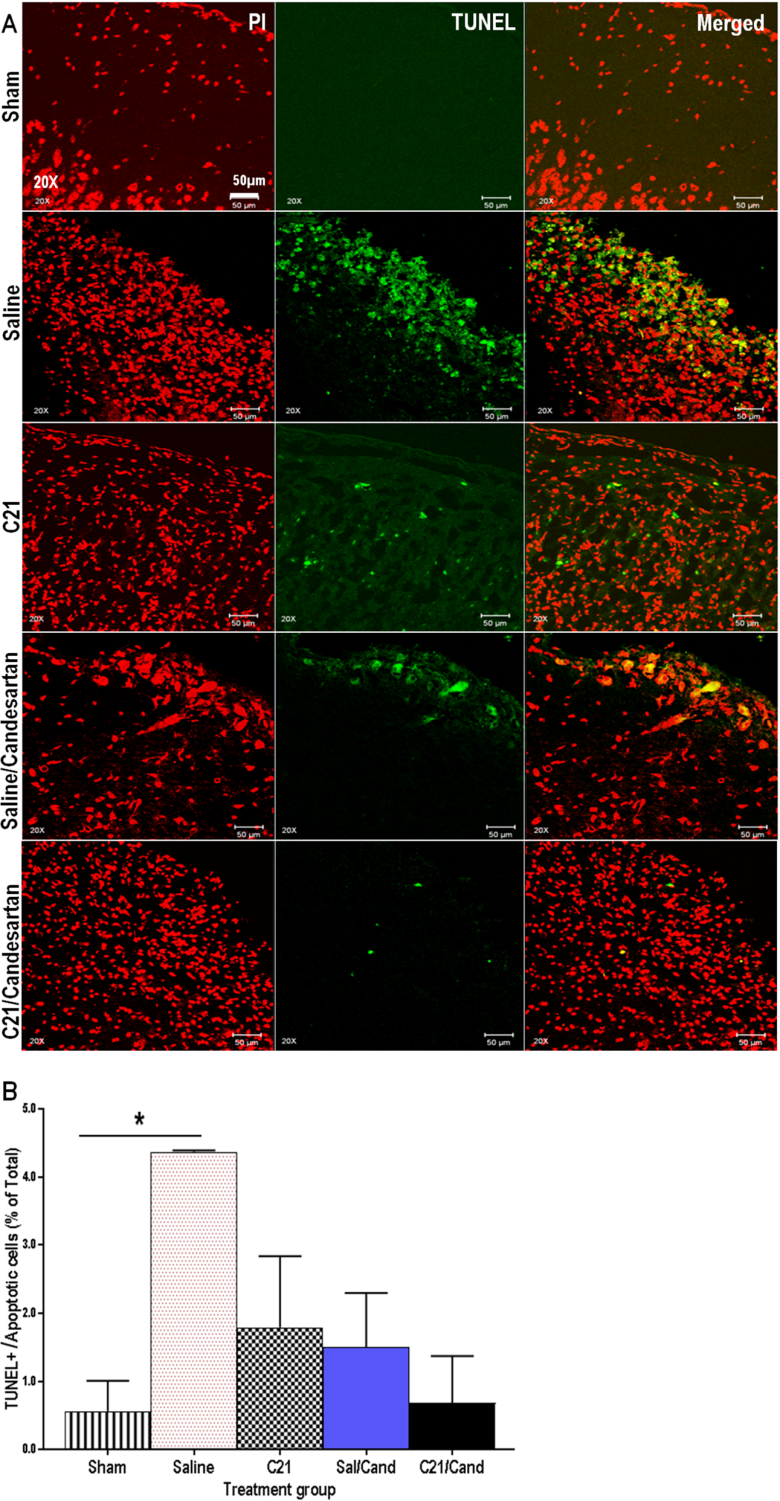
RAS modulation reduced apoptotic cell death post-stroke. **a** Representative 20× immunofluorescent images depicting TUNEL positive (green stained) cortical cells with red propidium iodide nuclear staining, scale bar 50 μm. **b** Animals subjected to tMCAO had a significantly greater number of tunnel positive cells in the ischemic hemisphere than shams *F*_(4,7)_ = 4.373, *P* = 0.0437. Cell death was greatly reduced in animals treated long-term with C21 and candesartan (*n* = 3–4 animals/group, error bars indicate SEM). Statistical significance for post hoc comparisons between groups using Tukey’s multiple comparison procedure are denoted by *P < 0.05 to indicate a difference from unstroked/sham animals

Animals subjected to tMCAO had significantly more TUNEL positive cells in their ischemic hemisphere than unstroked (sham) animals (Fig. 6a, b). Cell death was greatly reduced in animals treated long-term with C21/candesartan.

### RAS modulation reduced infarct/cavitation size in SHRs post-stroke

Several weeks after ischemia/reperfusion injury, a focal infarction typically develops into a cavitation with a unilateral loss of tissue in the infarct region [34, 35]. Due to the long-term nature of our study, the infarct area was replaced with a discrete cavitation in the ipsilateral hemisphere. This is difficult to accurately quantify due to collapsing of the tissue upon collection. For the brains where it was possible, we provide the data, which was quantified using ImageJ software (NIH) and calculated as percent of contralesional hemisphere (Additional file 5), but we believe it may not accurately reflect the drug effects. We have hence chosen functional outcomes as our primary endpoint.

## Discussion

The most important finding of our investigation was that chronic administration of RAS modulators, initiated after stroke, effectively prevented the development of cognitive impairment in hypertensive animals. This was true even when treatment was delayed to 7 days post-stroke and appeared to be independent of Aβ accumulation and blood pressure effects. This was consistent across a series of blinded tests, assessing different aspects of cognitive function. The C21 and candesartan-treated animals showed preserved short-term working memory at 21 and 24 days post-stroke. These animals also displayed better associative learning and reference memory at 28 days post-stroke, compared to saline-treated animals, despite a lack of difference in sensorimotor recovery. We are the first to report this late therapeutic window for RAS modulation and the prevention of post-stroke cognitive impairment. Regarding the mechanism of this protection, our results showed that RAS modulators, both C21 and candesartan, maintained microglial salutary resting state and prevented the pathologically exaggerated microglial inflammatory response that otherwise persists in hypertensive animals post-stroke. This was also associated with reduced amyloid accumulation in the brains of C21-treated animals and less apoptotic cell death with both RAS modulators.

Both animal [6, 36, 37] and human [5] studies demonstrate an association between RAS modulation and preservation of cognitive function, with a wide range of potential mechanisms. These agents not only block brain AT1R but also indirectly stimulate AT2R signaling, a specific target of C21, hence promoting its protective effects. ARBs are also effective at preventing Aβ-induced cytotoxicity and vascular damage [8]. Although RAS modulation has been mechanistically linked to a reduction in Aβ_1–42_ accumulation in studies of cognitive impairment [38, 39], our findings suggest other mechanisms of cognitive protection, so we investigated effects on cytotoxicity, apoptosis, and chronic inflammation.

C21, when administered for 7 days after ischemic stroke and either continued or replaced with low-dose candesartan, prevented accumulation of Aβ_1–42_ in the hippocampus and ischemic borderzone of SHRs post-stroke, but animals whose treatment was delayed, were started on saline for the first 7 days post-stroke, displayed cognitive preservation despite elevations in hippocampal Aβ_1–42_ at 30 days post-stroke. It has already been established that SHRs subjected to tMCAO tend to have Aβ accumulation of the ipsilateral cerebral cortex and hippocampus [40], but this is the first study to report the effect of C21 on brain Aβ_1–42_ concentration post-stroke.

It is possible, however, that RAS modulators, candesartan and C21, prevent cognitive decline by reducing Aβ_1–42_-mediated cytotoxicity. In hCMEC/D3, both doses of candesartan were equally effective at preventing Aβ_1–42_-associated endothelial cell death under hypoxic conditions and only the higher dose of C21 was effective under normoxic conditions. Aβ damages both endothelial and neuronal cells by oxidative stress and free-radical generation and activates a variety of inflammatory and pro-apoptotic pathways, all of which may play an important role in the pathogenesis of AD (Additional file 6) [15, 41]. Accordingly, we conducted our dose-response studies with Aβ_1–42_ concentrations in the nanomolar range (10–1000 nM) and chose the minimum toxic concentration (100 nM) for our cell culture studies. We also included OGD in order to simulate the ischemic conditions in the presence and absence of Aβ_1–42_. The combination of Aβ and cerebrovascular disease, including either chronic cerebral hypoperfusion and/or acute ischemia, has been shown to negatively affect cognition [42].

RAS modulators have also been shown to prevent chronic reactive microgliosis, otherwise associated with ischemia-reperfusion in hypertensive animals. Conversion of microglia from the normal resting state (ramified phenotype) to an activated state (amoeboidal phenotype) is the initial step in the CNS inflammatory response [40]. This inflammatory response, which develops in the brain over a period of hours to days after the onset of cerebral ischemia, may impair neurogenesis and other reparative processes essential to functional recovery after stroke. Microglia can become chronically activated by a single stimulus (ischemic stroke) to result in cumulative neuronal loss with time [43], similar to the time frame we observed in the emergence of cognitive decline. Moreover, a sustained activation of microglia is one of the first pathological features of neurodegenerative disease and cognitive impairment, long before there is any evidence of actual neurodegeneration [38]. In fact, it was recently determined that microglia actively contribute to the neuronal loss observed in animal models of AD [44]. Moreover, elimination of microglia in these animals resulted in a significant recovery of contextual memory and overall cognitive function with a reversal of dendritic spine loss and prevention of neuronal loss, without any changes in amyloid-β levels or plaque loads [44]. Chronic microglial stimulation is associated with a perpetual pro-inflammatory, pro-oxidative state that causes neurotoxicity and eventually results in cognitive decline [44]. In fact, overactivation of microglia is solely capable of inducing neuronal death in the absence of other pathological stimulation [45]. With other studies, treatment is almost always initiated at very short time points after the onset of ischemia. This prevents any mechanistic distinction between agents that directly suppress microglial activation with those that simply reduce inflammation only as a result of reduced cell injury. This is a considerable strength of our study since although our findings are in line with others showing that RAS modulators suppress microglial activation, we were the first to show this to be true long-term and even when treatment is delayed.

Our findings are also consistent with recent reports of immune-mediated delayed cognitive impairment in a mouse model of experimental stroke [46]. Investigators demonstrated that the cognitive decline appearing at least 7 days after stroke could be prevented by depleting or deactivating B-lymphocytes [46]. It is possible that the increased reactive microglia that we report could be involved in the homing of B-lymphocytes to the brain, further worsening cognitive decline.

Clinical trial evidence strongly suggests that BP lowering with candesartan in the acute stroke period (first 7 days) is associated with worse stroke outcomes [47]. Therefore, this investigation was a randomized, double-blind, preclinical trial, designed to mimic how C21 and candesartan might be prescribed to stroke patients.

We found that C21 does not affect the BP of hypertensive animals, when administered systemically, in multiple doses, either before or immediately after tMCAO. These results are similar to what we and others have seen in normotensive and hypertensive animals in doses of up to 10 mg/kg/day [8, 43, 44, 48–50]. Another important finding of this study was that acute ischemic stroke increases the sensitivity of hypertensive animals to the blood pressure lowering effects of candesartan. In fact, a low dose of candesartan (0.3 mg/kg), even at 7 days after stroke onset, resulted in an average BP reduction of ≈ 20 mmHg, similar to that achieved with the higher dose (1 mg/kg/day) of candesartan in unstroked animals. This is consistent with what we had previously reported in our study showing that hypertensive animals were more sensitive to a single dose of candesartan, administered immediately after stroke, than normotensive rats [20]. This may indicate that restarting blood pressure medications in patients post-stroke may be dangerous if the same pre-stroke doses are used, even when therapy is delayed to 7 days post-stroke.

We included sham animals to demonstrate the natural history of cognitive changes over 28 days in these young adult, hypertensive animals. The fact that this group did not demonstrate impairment in the NOR and the PAT support the notion that the significant cognitive decline in the saline-treated stroked animals at 28 days was due to the stroke injury. It is likely that sham hypertensive animals treated with either C21 or candesartan would also exhibit preserved cognition upon chronic administration, as has been demonstrated by others [7, 44]. Since we were unable to detect significant decline in our untreated shams at 28 days, however, a longer period of follow-up may be required for future studies.

## Conclusion

Our results clearly indicate that AT2R stimulation, either with the selective AT2R agonist C21, or the ARB, candesartan, may be beneficial in preventing the development of cognitive impairment after stroke, even when treatment is delayed to 7 days. This has important therapeutic implications and suggests therapy with C21 may be a valid approach for preserving cognition in various subsets of patients including certain elderly patients, those with severe carotid occlusive disease, and individuals with heart valve disorders, atrial fibrillation, and orthostatic hypotension. Although it is reasonable to withhold ARBs/BP lowering drugs during the acute period post-stroke, it may be appropriate and even advantageous to incorporate the novel, direct acting AT2 receptor agonist C21, as “bridge therapy,” until antihypertensive therapy initiation/re-initiation. This would exploit the numerous protective benefits associated with AT2R stimulation, and hence sustain cognitive function, without lowering blood pressure. It may also be used in place of agents like candesartan in those with allergies or intolerance to other RAS modulators and are being treated with non-ACE/ARB blood pressure medications.

## Additional files

Additional file 1: C21 ameliorates weight loss. C21 when administered daily, starting at 2 h after tMCAO and reperfusion, significantly ameliorated weight loss at day 7, compared to saline-treated controls, with animals showing most rapid recovery, between days 7 and 30, when treatment was followed with candesartan. (TIF 246 kb)

Additional file 2: Cognition declines over time after stroke. Despite complete recovery of sensorimotor function, the saline-treated animals demonstrated a continuous decline in cognition from baseline to day 14 to day 21. Statistical significance denoted by *P < 0.0001 for an effect of time, while **P < 0.01 indicates significance between the 2 treatment groups (C21 vs. saline), effect of treatment at 21 days post-stroke, and #*P* < 0.005 indicates presence of a significant interaction between time and treatment effects. (TIF 123 kb)

Additional file 3: C21 reduces Aβ accumulation in the cortex after stroke. ELISA analysis showed that animals treated with C21, for the first 7 days, had markedly lower concentrations of Aβ_1–42_ in their cortical ischemic borderzones, at 30 days post-stroke compared to those treated with saline. Statistical significance for post hoc comparisons between groups using Tukey’s multiple comparison procedure are denoted by *P < 0.01 to indicate a difference from saline. (TIF 149 kb)

Additional file 4: RAS modulators reduced the total volume of injury after stroke. RAS modulation reduced infarct/cavitation size in SHRs post-stroke. The sections were stained with hematoxylin and eosin (H&E) stain and infarct/cavitation volumes quantified and expressed as a percentage of the contralateral side. Statistical significance for post hoc comparisons between groups using Tukey’s multiple comparison procedure are denoted by *P < 0.01 to indicate a difference from all other treatment groups. (TIF 722 kb)

Additional file 5: RAS modulation reduces neuronal cytotoxicity. Cell viability was significantly reduced in primary neurons incubated with Aβ_1–42_ compared with untreated controls, under similar conditions. This Aβ_1–42_-mediated cytotoxicity was reduced when cells were co-treated with C21. This reached statistical significance only with the higher dose of C21. Statistical significance for post hoc comparisons between groups using Tukey’s multiple comparison procedure are denoted by *P < 0.05 to indicate a viability substantially higher than that seen for Aβ_1–42_-treated cells. (TIF 155 kb)

Additional file 6: Conceptual diagram. Acute cerebral hypoperfusion and hypoxia, due to stroke and/or chronic cerebral hypoperfusion, facilitates amyloid-β (Aβ) production by activating β-secretase enzyme, necessary for Aβ production. Aβ is a potent vasoconstrictor that worsens cerebral hypoperfusion by further reducing cerebral blood flow (CBF) and hence transvascular transport, thereby reducing its own clearance leading to additional accumulation and toxicity. Aβ also results in microglial activation, neuroinflammation, and endothelial dysfunction. It creates a vicious cycle whereby the endothelial dysfunction, which is associated with diminished vasodilation, leads to further reductions in cerebral blood flow (CBF) and hypoperfusion, BBB disruption, and alterations in permeability resulting in tissue edema which further reduces CBF, by compressing blood vessels. The resulting hypoperfusion leads to additional oxidative stress by inducing tissue hypoxia. The oxidative and pro-inflammatory environment induced by hypoperfusion and BBB breakdown results in demyelination, synaptic defects, and disruption of trophic coupling between neurovascular unit (NVU) components. Therefore, vascular dysfunction and stroke are tightly linked to Aβ accumulation and neuronal dysfunction. They are ultimately manifested as vascular cognitive impairment/dementia (VCID). Stimulation of the AT2R by C21 acts to reduce Aβ accumulation and possesses neurovascular benefits by virtue of its potent anti-inflammatory, antioxidant, and tissue-regenerating properties. ★ Pathways targeted by C21. (TIF 3845 kb)

## Abbreviations

AD: Alzheimer’s disease
Ang II: Angiotensin II
ARBs: Angiotensin II type 1 receptor blockers
AT1R: Ang II type 1 receptor
AT2R: Ang II type 2 receptors
BDNF: Brain-derived neurotrophic factor
C21: Compound 21
CBF: Cerebral blood flow
ELISA: Enzyme linked immunosorbent assay
FDA: Food and Drug Administration
GAPDH: Glyceraldehyde 3-phosphate dehydrogenase
HBECs: Human brain endothelial cells
IACUC: Institutional Animal Care and Use Committee
IP: Intraperitoneal
NGF: Nerve growth factor
NIH: National Institute of Health
NOR: Novel object recognition
OGD: Oxygen glucose deprivation
PAT: Passive avoidance tests
RAS: Renin angiotensin system
SAP: Spontaneous alternation test
SHRs: Spontaneously hypertensive rats
tMCAO: Transient middle cerebral artery occlusion
VCID: Vascular cognitive impairment/dementia
