# Supplementary figures and images for: RAS modulation prevents progressive cognitive impairment after experimental stroke: a randomized, blinded preclinical trial
Source: J Neuroinflammation. 2018 Aug 13;15:229. doi: 10.1186/s12974-018-1262-x (PMC6090822; doi:10.1186/s12974-018-1262-x)

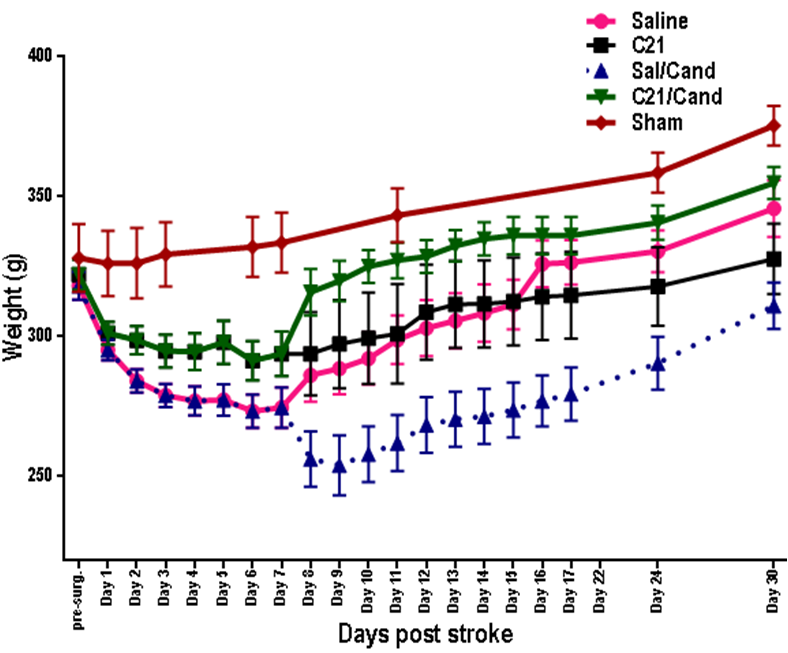

Supplement: Supplementary file 1 — C21 ameliorates weight loss. C21 when administered daily, starting at 2 h after tMCAO and reperfusion, significantly ameliorated weight loss at day 7, compared to saline-treated controls, with animals showing most rapid recovery, between days 7 and 30, when treatment was followed with candesartan. (TIF 246 kb) [file 12974_2018_1262_MOESM1_ESM.tif]

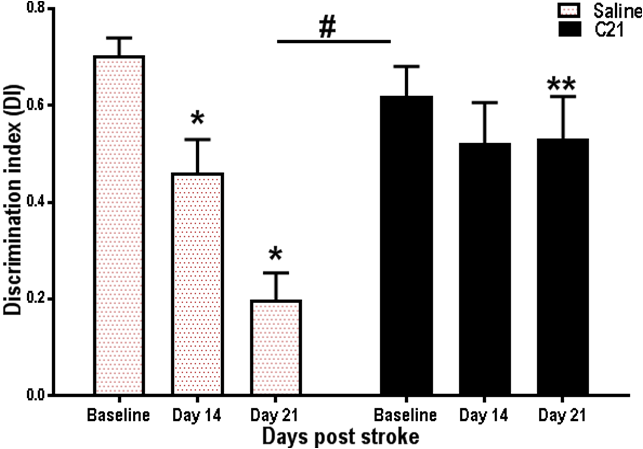

Supplement: Supplementary file 2 — Cognition declines over time after stroke. Despite complete recovery of sensorimotor function, the saline-treated animals demonstrated a continuous decline in cognition from baseline to day 14 to day 21. Statistical significance denoted by *P < 0.0001 for an effect of time, while **P < 0.01 indicates significance between the 2 treatment groups (C21 vs. saline), effect of treatment at 21 days post-stroke, and #P < 0.005 indicates presence of a significant interaction between time and treatment effects. (TIF 123 kb) [file 12974_2018_1262_MOESM2_ESM.tif]

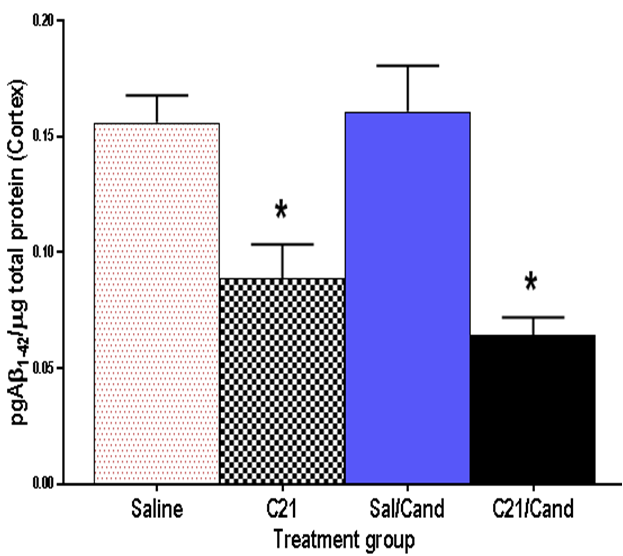

Supplement: Supplementary file 3 — C21 reduces Aβ accumulation in the cortex after stroke. ELISA analysis showed that animals treated with C21, for the first 7 days, had markedly lower concentrations of Aβ1–42 in their cortical ischemic borderzones, at 30 days post-stroke compared to those treated with saline. Statistical significance for post hoc comparisons between groups using Tukey’s multiple comparison procedure are denoted by *P < 0.01 to indicate a difference from saline. (TIF 149 kb) [file 12974_2018_1262_MOESM3_ESM.tif]

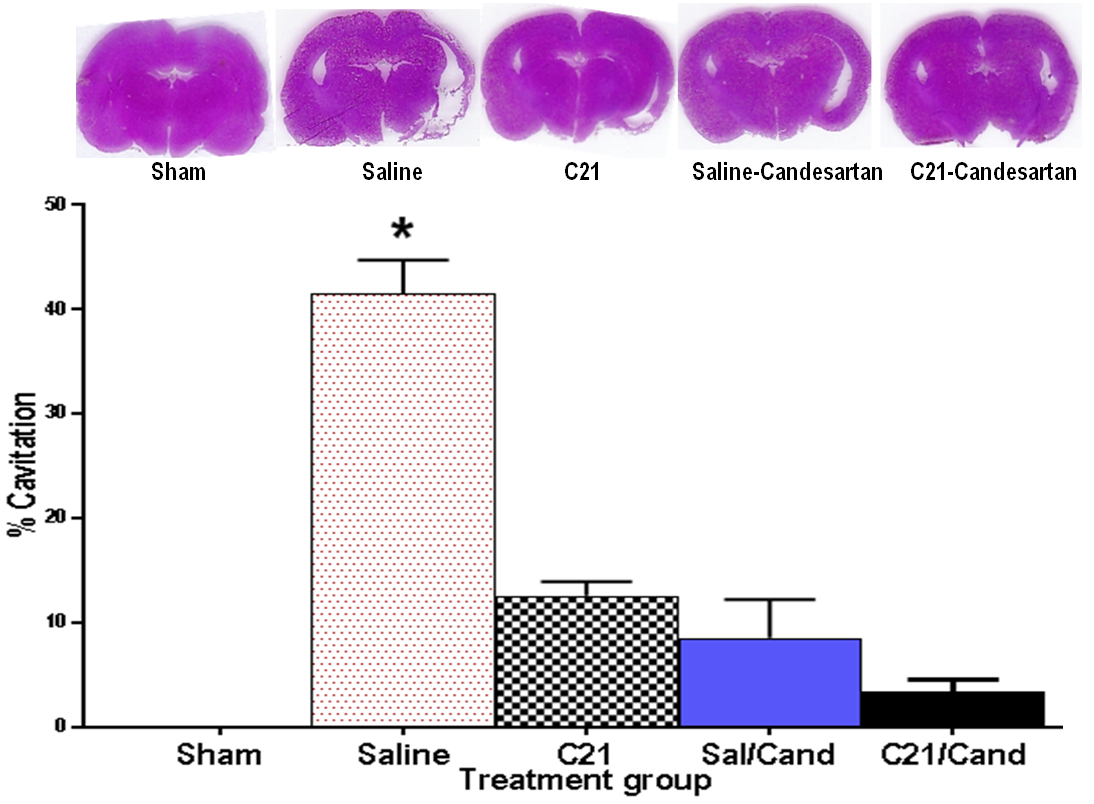

Supplement: Supplementary file 4 — RAS modulators reduced the total volume of injury after stroke. RAS modulation reduced infarct/cavitation size in SHRs post-stroke. The sections were stained with hematoxylin and eosin (H&E) stain and infarct/cavitation volumes quantified and expressed as a percentage of the contralateral side. Statistical significance for post hoc comparisons between groups using Tukey’s multiple comparison procedure are denoted by *P < 0.01 to indicate a difference from all other treatment groups. (TIF 722 kb) [file 12974_2018_1262_MOESM4_ESM.tif]

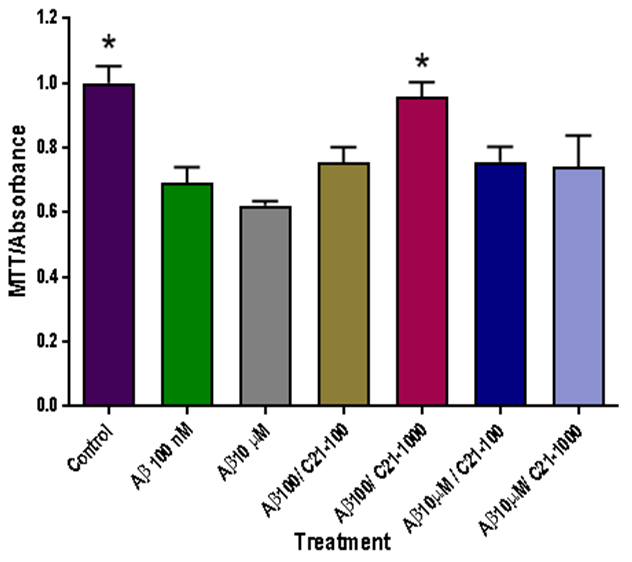

Supplement: Supplementary file 5 — RAS modulation reduces neuronal cytotoxicity. Cell viability was significantly reduced in primary neurons incubated with Aβ1–42 compared with untreated controls, under similar conditions. This Aβ1–42-mediated cytotoxicity was reduced when cells were co-treated with C21. This reached statistical significance only with the higher dose of C21. Statistical significance for post hoc comparisons between groups using Tukey’s multiple comparison procedure are denoted by *P < 0.05 to indicate a viability substantially higher than that seen for Aβ1–42-treated cells. (TIF 155 kb) [file 12974_2018_1262_MOESM5_ESM.tif]

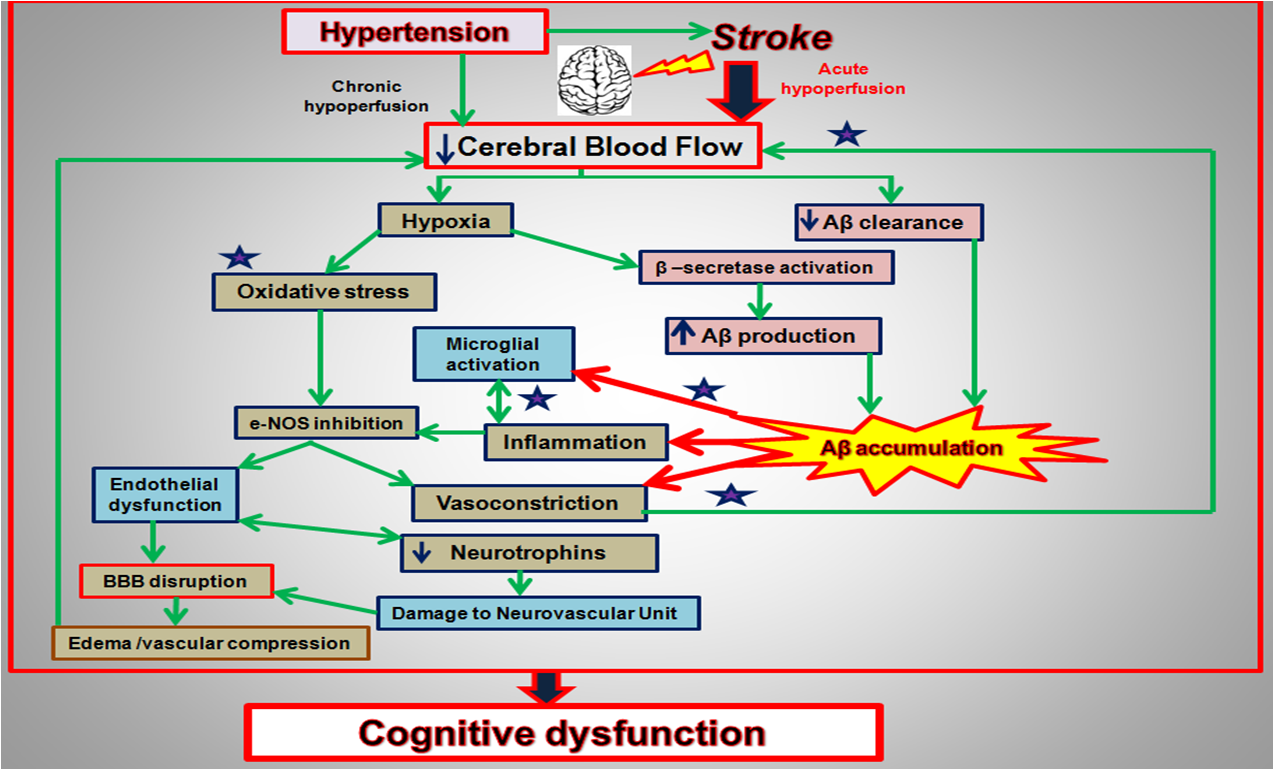

Supplement: Supplementary file 6 — Conceptual diagram. Acute cerebral hypoperfusion and hypoxia, due to stroke and/or chronic cerebral hypoperfusion, facilitates amyloid-β (Aβ) production by activating β-secretase enzyme, necessary for Aβ production. Aβ is a potent vasoconstrictor that worsens cerebral hypoperfusion by further reducing cerebral blood flow (CBF) and hence transvascular transport, thereby reducing its own clearance leading to additional accumulation and toxicity. Aβ also results in microglial activation, neuroinflammation, and endothelial dysfunction. It creates a vicious cycle whereby the endothelial dysfunction, which is associated with diminished vasodilation, leads to further reductions in cerebral blood flow (CBF) and hypoperfusion, BBB disruption, and alterations in permeability resulting in tissue edema which further reduces CBF, by compressing blood vessels. The resulting hypoperfusion leads to additional oxidative stress by inducing tissue hypoxia. The oxidative and pro-inflammatory environment induced by hypoperfusion and BBB breakdown results in demyelination, synaptic defects, and disruption of trophic coupling between neurovascular unit (NVU) components. Therefore, vascular dysfunction and stroke are tightly linked to Aβ accumulation and neuronal dysfunction. They are ultimately manifested as vascular cognitive impairment/dementia (VCID). Stimulation of the AT2R by C21 acts to reduce Aβ accumulation and possesses neurovascular benefits by virtue of its potent anti-inflammatory, antioxidant, and tissue-regenerating properties. ★ Pathways targeted by C21. (TIF 3845 kb) [file 12974_2018_1262_MOESM6_ESM.tif]
